# Deep learning-radiomics-SUVmax integration from ^18^F-FDG PET/CT predicts synchronous distant metastasis in nasopharyngeal carcinoma

**DOI:** 10.3389/fonc.2026.1728616

**Published:** 2026-08-26

**Authors:** Yun Zhang, Ying Cao, Shanshan Xu, Yan Tang, Jian He, Jing Chen, Ziwei Nie, Yuxiao Hu

**Affiliations:** 1 Department of PET/CT Center, Jiangsu Cancer Hospital& Jiangsu Institute of Cancer Research & The Affiliated Cancer Hospital of Nanjing Medical University, Nanjing, China; 2 School of Mathematics, Nanjing University, Nanjing, China; 3 Department of Nuclear Medicine, Nanjing Drum Tower Hospital, The Affiliated Hospital of Nanjing University Medical School, Nanjing, China

**Keywords:** 18F-FDG PET/CT, deep learning, hybrid model, nasopharyngeal carcinoma, synchronous distant metastases

## Abstract

**Objectives:**

This study aimed to develop and evaluate deep learning (DL) models of primary tumor (T) and cervical metastatic lymph nodes (CMLNs) using fluorine-18 fluorodeoxyglucose positron emission computed tomography (^18^F-FDG positron emission tomography/computed tomography (PET/CT)) imaging for predicting synchronous distant metastasis (SDM) in nasopharyngeal carcinoma (NPC) patients, integrating radiomic features, primary tumor maximum standardized uptake value (SUVmax-T) and clinical features.

**Methods:**

A two-center retrospective cohort of 218 patients (105 SDM, 113 non-SDM) was analyzed. Three DL architectures (ResNet18, DenseNet121, EfficientNet-B0) were trained on CT-only, PET-only, and fused CT+PET images. Nine models combining DL features, radiomic features of T + CMLNs or T-only, clinical data, and SUVmax-T using Multilayer Perceptron (MLP) and Random Forest (RF) classifiers were established. Multiple MLP benchmark models were built based on separate T/N staging, clinical data, SUVmax-T and deep features for comparative analysis. Performance was assessed via accuracy, precision, recall, F1-score, and ROC-AUC. Permutation feature importance analysis identified core predictors of the optimal RF model, and the optimal hybrid model underwent external multicenter validation.

**Results:**

Dual-modal PET/CT networks surpassed single-modality models, with ResNet18 yielding the highest internal ROC-AUC of 0.804, superior to DenseNet121 (0.791) and EfficientNet-B0 (0.779). Integrating radiomic features (T), clinical parameters and SUVmax-T continuously elevated model discrimination, and the MLP hybrid model reached the peak internal ROC-AUC of 0.839 (recall = 0.747), outperforming the RF model (ROC-AUC = 0.787, recall = 0.777). Models built only on T/N staging reached an internal AUC of 0.476 and an external AUC of 0.513, while integrating deep features boosted their predictive AUC to 0.658 (internal) and 0.688 (external). External validation of the MLP hybrid model showed an ROC-AUC of 0.701 and a PR-AUC of 0.571. SUVmax-T was identified as the most important predictive feature in the RF hybrid model.

**Conclusion:**

Integrating ResNet18 (CT+PET) features with radiomic features (T), SUVmax-T and clinical data in an MLP framework yielded the highest performance, with SUVmax-T identified as an SDM predictor. Comparative benchmark analysis verified that this multimodal hybrid model achieved better discriminative performance than models relying solely on conventional T and N staging.

## 1 Introduction

Globally, nasopharyngeal carcinoma (NPC) accounts for 0.7% (133,354 cases) of new cancer cases and 0.8% (80,008 deaths) of cancer-related mortality. NPC is projected to rise significantly in incidence and mortality by 2040, particularly in Asian populations (1, 2). Owing to the anatomically concealed location of the nasopharynx, 4-10% of NPC patients present with distant metastases (DM) at initial diagnosis (3).

Released in 2025, the 9th AJCC/UICC TNM staging system for nasopharyngeal carcinoma (NPC) includes two major revisions to refine risk stratification compared with the 8th edition. First, radiologic extranodal extension of cervical lymph nodes is recognized as an adverse prognostic factor and categorized as stage N3 to identify patients with aggressive locoregional disease. Second, the original M1 distant metastasis category in the 8th edition is subdivided into M1a (≤3 metastatic lesions across all organs) and M1b (>3 lesions), enabling differentiated curative or palliative therapeutic strategies for patients with metastatic NPC (4).

M1-stage distant metastases are further classified into synchronous distant metastases (SDM) and metachronous distant metastases (MDM) (5). SDM is defined as distant metastasis detected at initial NPC diagnosis or within three months after radiotherapy, while MDM refers to metastasis occurring more than three months after definitive radiotherapy. The presence of occult SDM fundamentally changes the overall treatment strategy: patients confirmed to have SDM cannot receive radical local radiotherapy alone and need upfront systemic anti-tumor therapy, while patients without baseline metastatic lesions are eligible for curative radiotherapy as the core treatment. Therefore, accurate identification of hidden SDM before initial treatment is the key basis for formulating individualized clinical management plans. Although numerous studies have investigated MDM in NPC (6–13), research focusing specifically on SDM remains scarce. In contrast, SDM-related predictive and prognostic analyses have been extensively conducted in various solid tumors, including lung adenocarcinoma (14), pancreatic ductal adenocarcinoma (15), breast cancer (16), gallbladder cancer (17), and gastric cancer (18).

Pretreatment identification and quantitative evaluation of SDM are essential for individualized therapeutic decision-making. As the mainstream imaging modality for systemic staging, ¹^8^F-FDG PET/CT exhibits high sensitivity and specificity for SDM detection with a low false-positive rate, outperforming conventional staging approaches (19–22). Nevertheless, approximately 18% false-negative cases persist in routine clinical practice, representing a major obstacle for accurate baseline risk stratification (21).

Previous studies have attempted to explore SDM-related risk factors based on molecular mechanisms (23–25), yet few have incorporated tumor metabolic characteristics. Notably, ¹^8^F-FDG PET/CT provides robust quantitative metabolic biomarkers that reflect underlying tumor biological behavior (26–29).

Advancements in medical image analysis have facilitated high-throughput quantitative feature extraction, promoting the clinical application of radiomics by converting medical images into mineable high-dimensional data to assist clinical decision-making (30). In the field of NPC, radiomics has been increasingly applied for preoperative diagnosis, therapeutic response evaluation, and prognostic stratification (31).

With the rapid development of artificial intelligence, deep learning (DL) algorithms have demonstrated powerful capability in mining implicit information from medical images. DL-based models have been widely implemented in NPC for preoperative diagnosis, recurrence and metastasis detection, and prognostic prediction (12, 32–37).

Conventional anatomical TNM staging is partially predictive of SDM risk, as advanced T classification and extensive cervical lymph node involvement correlate with elevated metastatic likelihood (4). However, this morphology-only staging framework cannot quantify intratumoral glycolytic heterogeneity that governs metastatic propensity. Even among patients with identical T and N grades, metastatic risk varies substantially. Handcrafted radiomics, a classic quantitative imaging approach, carries intrinsic drawbacks. It depends on fixed predefined feature pools and cannot automatically capture high-order multimodal anatomical and metabolic signatures associated with tumor metastatic potential. In contrast, existing DL models for NPC metastasis predominantly focus on post-treatment MDM prediction based solely on MRI data. DL algorithms can hierarchically capture latent multimodal features from raw PET/CT data, effectively compensating for the inherent deficiencies of anatomy-only TNM staging and predefined radiomic frameworks.

This study addresses a critical research gap. Existing DL models for NPC metastasis predominantly focus on post-treatment MDM prediction based solely on MRI data (12, 38). To date, few studies have established bimodal ¹^8^F-FDG PET/CT DL frameworks for baseline occult SDM screening in NPC. SDM occurs in only a small proportion of treatment-naïve NPC patients at initial diagnosis, which inherently limits the enrollment of sufficient SDM-positive cases in retrospective observational cohorts.

As a preliminary imaging study, the present study aimed to develop a pretreatment DL model integrating multimodal features derived from both primary tumors (T) and cervical metastatic lymph nodes (CMLNs) using dual-modal PET/CT data for individualized baseline SDM risk prediction. Given that radiomic and metabolic parameters have been proven to complement and enhance DL model performance in NPC prognostic evaluation (39), this study further explored whether the integration of T- and CMLNs-derived multimodal features could optimize the diagnostic efficacy of DL-based SDM prediction. The established model is expected to serve as an auxiliary pretreatment risk-stratification tool for identifying high-risk NPC patients requiring intensified clinical verification, thereby facilitating individualized pretreatment staging and surveillance strategies.

## 2 Materials and methods

### 2.1 Patient cohorts

This retrospective multicenter study was approved by the Institutional Ethics Review Boards of Jiangsu Cancer Hospital and Nanjing Drum Tower Hospital. The requirement for informed consent was waived because of the retrospective nature of the study.

#### 2.1.1 Internal dataset

The internal cohort consisted of 193 NPC patients (94 with SDM, 99 without SDM). All subjects were retrospectively selected from 1928 treatment-naive NPC patients receiving ¹^8^F-FDG PET/CT at Jiangsu Cancer Hospital from April 2017 to October 2024. The inclusion criteria were as follows: (1) age ≥18; (2) diagnosis of squamous cell carcinoma by endoscopic biopsy; (3) all participants were reassigned to the 9th AJCC/UICC TNM staging system. All patients with original staging recorded under the 8th TNM edition were retrospectively re-evaluated by two experienced radiation oncologists according to the 9th edition criteria to unify risk evaluation standards; (4) SDM was confirmed either immediately by these initial scans or through subsequent imaging assessments. If immediate supplementary imaging was unavailable or returned negative findings, patients received quarterly follow-up scans for at least 12 months. Lesions with stable size during surveillance were regarded as locoregional lesions, whereas progressive lesions were defined as SDM (5, 12). The exclusion criteria were as follows: (1) The lesion in the nasopharynx was not complete in PET images; (2) PET images could not be converted to SUV values; (3) other malignant neoplasms.

#### 2.1.2 External test set

**Figure 1:**
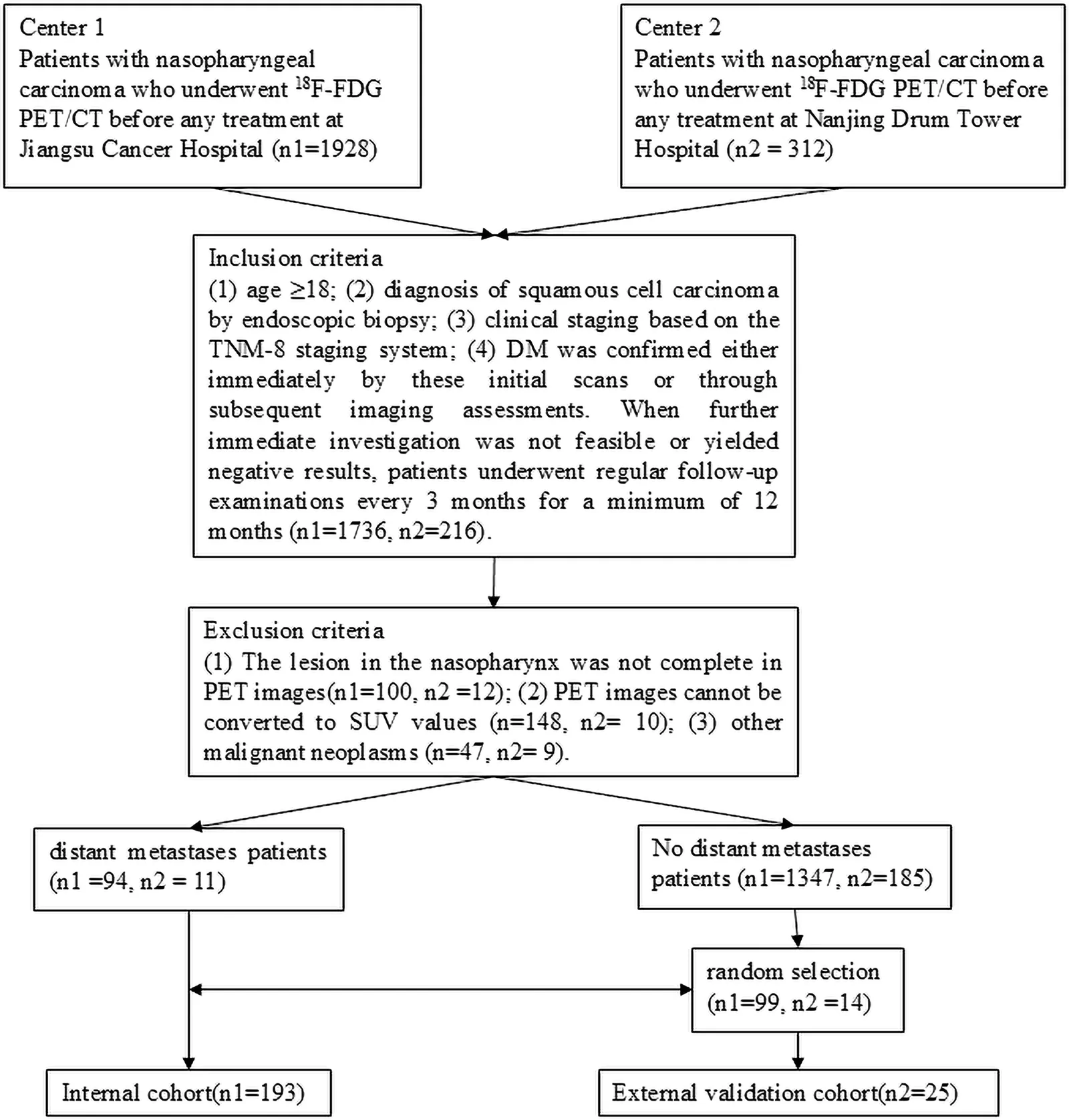
Flow diagram of patient selection in the internal cohort, and external validation cohort.

The external test set contained 25 NPC patients (11 SDM, 14 non-SDM) from 312 NPC patients from Nanjing Drum Tower Hospital between December 2014 and October 2023. A flow diagram of patient selection is shown in **Figure 1**.

### 2.2 Image acquisition

Patients underwent ^18^F-FDG PET/CT on a Discovery 710 scanner (GE Healthcare) at Jiangsu Cancer Hospital or a Philips Vereos 64 PET/CT (Philips) at Nanjing Drum Tower Hospital. Following ≥ 6 h fasting and confirmation of blood glucose < 11 mmol/L, patients received 3.7–7.4 MBq/kg ¹^8^F-FDG intravenously. After 50–70 min uptake (resting, oral hydration ~1000 mL, voiding pre-scan), vertex-to-mid-thigh imaging commenced.

Jiangsu Cancer Hospital: CT: 140 kV, auto-mA (noise index 28.5), 0.8 s rotation, 3.75-mm slices. PET: 2 min/bed, 6–7 beds. Reconstruction: VUE point FX + SharpIR (2 iterations, 24 subsets) with CT-based attenuation correction.

Nanjing Drum Tower Hospital: CT: 120 kV, 80–120 mAs, 2/4-mm slices. PET: 1 min/bed, 8–10 beds. Reconstruction: OSEM (3 iterations, 10 subsets) with CT-based attenuation correction.

### 2.3 Data preprocessing

A systematic preprocessing pipeline standardized PET/CT image formats, enhanced data consistency, and optimized performance for subsequent DL classification in NPC patients with SDM. Using Python libraries (NiBabel, SimpleITK, MONAI), raw DICOM PET/CT images were converted to NIfTI (.nii.gz) format via NiBabel to facilitate processing.

#### 2.3.1 PET image SUV conversion

To quantify the radiotracer uptake intensity in PET images, pixel values were converted to standardized uptake values (SUV) based on metadata extracted from the DICOM headers. The SUV was calculated using the following formula:


$$\text{SUV}=\text{Pixel Value}\times \frac{\text{Patient Weight }(\text{g})}{\text{Injected Dose }(\text{Bq})}\times \text{Decay Correction Factor}$$

Pixel values were linearly corrected using the “Rescale Slope” and “Rescale Intercept” parameters from the DICOM headers, with the injected dose and decay correction factor also extracted from the metadata. This step ensured that PET image signal intensities were physiologically meaningful, enabling cross-patient comparisons.

#### 2.3.2 Spatial normalization and standardization

In the spatial preprocessing step, all PET and CT images were resampled to an isotropic voxel size of 1 × 1 × 1 mm^3^ using bicubic spline interpolation, and subsequently resized to the same matrix size for complete consistency across modalities. Following spatial alignment, a standardized three-dimensional cropping was performed to encompass the entire head-and-neck region, and all images were normalized to a uniform dimension of 512 × 512 × 80 voxels. The final cropped volumes were independently reviewed and confirmed by two experienced radiologists using 3D Slicer to guarantee that both the T and CMLNs were fully contained within the field of view.

#### 2.3.3 Volume of interest extraction

Two experienced radiologists independently delineated T (label 1) and CMLNs (label 2) in 3D using 3D Slicer. The two radiologists were blinded to the information of all the patients during the period of segmentation. Following semiautomatic segmentation, two radiologists manually adjusted slice-wise VOI borders of primary tumors (T) and cervical metastatic lymph nodes (CMLNs) to exclude adjacent normal tissues and generated binary lesion masks via SimpleITK. For DL pipelines, the input data consisted of 3D VOI volumes derived from CT and PET, with non-lesion regions assigned background values (CT: −1024, PET: 0). For radiomics feature extraction, features were extracted exclusively within binary lesion masks on spatially standardized images resampled to a uniform voxel spacing of 1 × 1 × 1 mm^3^ and a matrix size of 512 × 512 × 80 voxels, thereby eliminating signal interference from perilesional normal tissues.

#### 2.3.4 Training and validation set partitioning

We adopted stratified five-fold cross-validation to fully utilize limited patient samples. The cohort was split into five folds with balanced proportions of SDM-positive and SDM-negative patients in each fold, where four folds were used for training and one for validation per cycle.

### 2.4 Two-stage modelling pipeline

We proposed a two-stage PET/CT-based framework integrating multimodal DL and radiomic features, using clinical variables and SUVmax-T to predict SDM in NPC patients.

#### 2.4.1 Multimodal DL classification

Three Convolutional Neural Network (CNN) architectures—ResNet18 (skip connections) (40), DenseNet121 (dense connectivity) (41), and EfficientNet-B0 (compound scaling) (42)—were implemented for SDM prediction. This multi-model approach leveraged complementary feature extraction capabilities across three input modalities: CT-only, PET-only, and channel concatenated PET/CT. Models were trained with Adam and cross-entropy loss using five-fold cross-validation. Random online augmentation (flip, rotation, scaling) was integrated to mitigate overfitting caused by limited cohort scale.

#### 2.4.2 Model training and hyperparameter settings

All DL models were implemented in the MONAI framework (version 1.4. dev2430) based on PyTorch and executed on an NVIDIA A100 GPU (40 GB memory). Prior to model input, images were intensity-normalized (CT: HU values linearly mapped from −1000–400 to [0,1]; PET: SUV values mapped from 0–20 to [0,1]). Training employed the Adam optimizer with cross-entropy loss, an initial learning rate of 1e-4, and a batch size of 2 for CT-only, PET-only, and PET/CT fusion models. The maximum training epoch was set to 100, with the optimal epoch determined through cross-validation and used for final model evaluation on the external test set. To mitigate overfitting, data augmentation included random flipping along the x-axis (p = 0.5), random rotations ( ± 15° along x, y, z axes, p = 0.5), and random scaling (0.9–1.1, p = 0.5).

#### 2.4.3 Radiomic features extraction and multi-feature fusion

Stage two integrated radiomic features, clinical variables, and SUVmax-T into a multimodal fusion model. Machine learning models, including Random Forest (RF) and Multilayer Perceptron (MLP), were constructed using the Scikit-learn library (version 1.6.1). All experiments were conducted on a computing environment equipped with an NVIDIA A100 GPU with 40 GB of memory.

##### 2.4.3.1 Radiomics feature extraction

Radiomic features of primary tumors (T, label 1) and CMLNs (label 2) were extracted from CT and PET scans via PyRadiomics following IBSI standardized benchmarks. All images underwent built-in PyRadiomics normalization (normalize=True, normalizeScale=100), which rescaled voxel intensities to a zero mean and set the standard deviation to 100 for more stable feature outputs.

We acquired four classes of radiomic descriptors: shape metrics, first-order statistics, texture matrices, and wavelet-based high-order features. Each modality generated 1040 features, and we calculated these descriptors separately for T-only regions and combined T-CMLN regions, yielding 2080 features for each region type.

We retained PyRadiomics default settings with several customized parameters (binWidth = 25, sigma = [3, 5], resampledPixelSpacing = [1, 1, 1]). After extraction, all radiomic features were standardized individually using StandardScaler. The scaler was fitted exclusively on the training cohort, and the same transformation was directly applied to validation and test cohorts to maintain consistent preprocessing. Complete PyRadiomics parameter documentation is available at https://pyradiomics.readthedocs.io/en/latest/.

##### 2.4.3.2 Deep feature extraction and fusion

The best-performing DL model (ResNet-18) was used to extract a 512-dimensional feature vector from the layer preceding the fully connected layer. For feature-level fusion, PyRadiomics-derived radiomic features of the primary tumor +CMLNs were concatenated with 3D DL features from the dual-channel ResNet-18 model, and all features were standardized using Z-score normalization.

##### 2.4.3.3 Integration of clinical variables and metabolic parameters

Five clinical variables (age, gender, fasting blood glucose, height, weight) and SUVmax-T were standardized via StandardScaler and concatenated with fused deep-learning-radiomics signatures to generate the final input feature vector. RF and MLP models were trained on this combined feature set to predict the probability of SDM. The model achieving the highest internal AUC was selected for subsequent independent external multicenter validation.

To quantitatively verify whether our multimodal imaging biomarkers deliver incremental predictive value beyond routine anatomical T and N staging, we constructed a standalone MLP benchmark model. Categorical T and N stages were encoded via one-hot transformation as exclusive input features, while M-stage data (the predictive endpoint) were excluded to avoid circular logic. To guarantee fair performance comparison, this benchmark adopted identical network architecture, stratified five-fold cross-validation pipeline and data augmentation protocols as our primary hybrid model. We further established multiple intermediate combined feature subsets (T+N plus clinical- SUVmax-T features, T+N plus DL features) to disentangle the independent predictive contribution of each imaging component. Considering that manual T/N staging relies on subjective visual assessment and suffers from substantial inter-reader variability, categorical T and N staging variables were ultimately excluded from our final optimal model, which solely relies on objective quantitative imaging biomarkers automatically extracted from PET/CT volumes.

### 2.5 Feature importance analysis

SHAP (Shapley Additive Explanations) (43) analysis quantified contributions of clinical variables (age, gender, blood glucose, height, weight) and SUVmax-T to predictions. SHAP, grounded in game theory’s Shapley values, assigns feature contribution scores to individual predictions, providing global interpretability. TreeExplainer computed SHAP values for the RF model. Global feature importance was determined by mean absolute SHAP value.

**Figure 2:**
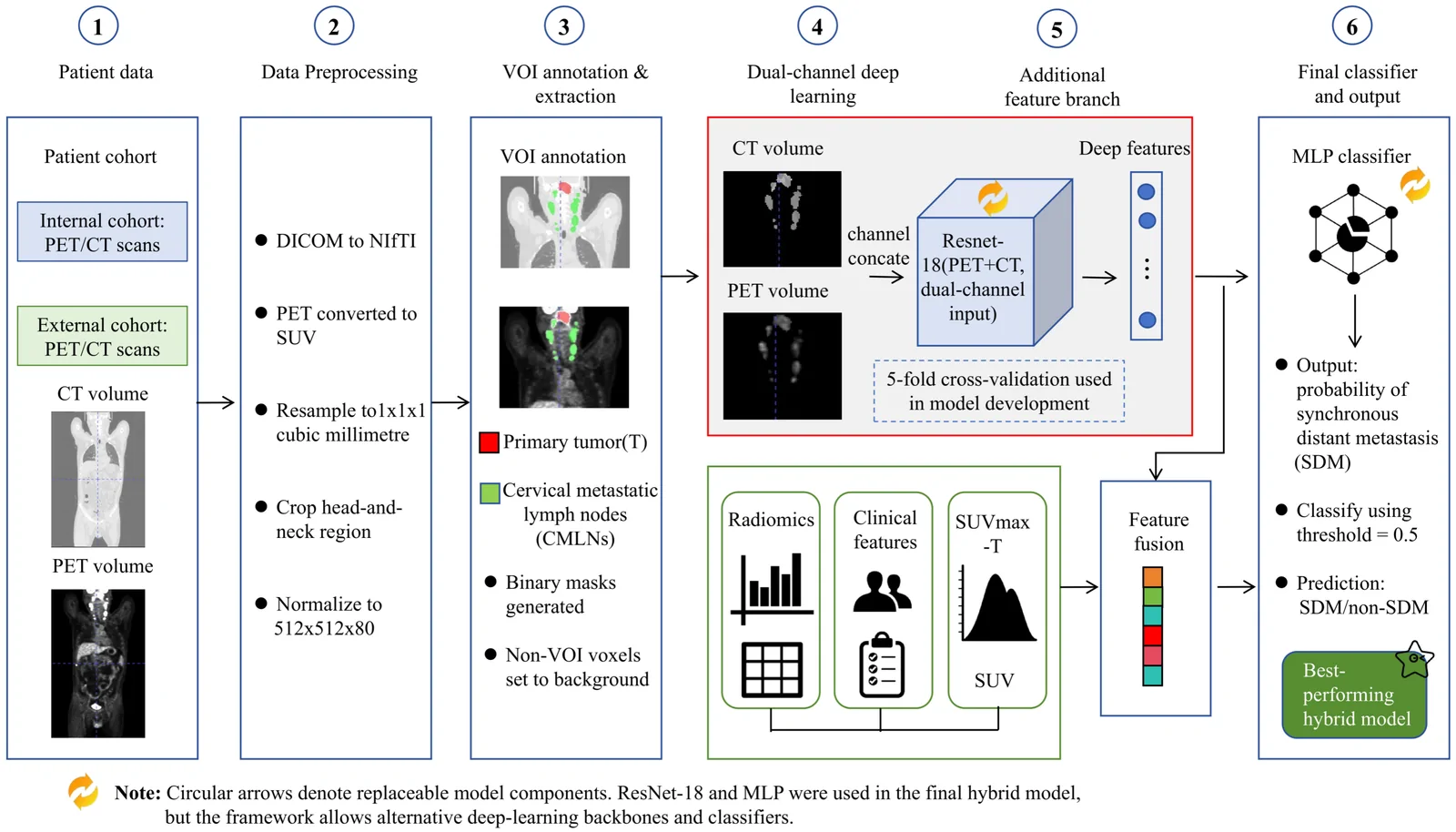
Workflow of the proposed PET/CT-based multimodal hybrid model for SDM prediction, including data preprocessing, dual-channel DL feature extraction, multi-source feature fusion and classification.

The overall construction pipeline of the two-stage multimodal hybrid prediction model is visualized in **Figure 2**.

### 2.6 Statistical analysis

Continuous variables were first tested for normality using the Shapiro-Wilk test. Normally distributed variables were presented as mean ± standard deviation and compared using independent t-tests, while non-normally distributed variables were presented as median (interquartile range) and compared using the Mann-Whitney U test. The chi-square test was used to compare categorical variables. A two-sided *p* value< 0.05 was defined as the threshold of statistical significance.

Intraclass correlation coefficients (*ICCs*) were calculated to quantify inter-reader reproducibility of extracted DL features, handcrafted radiomic features and SUVmax-T metabolic parameters between two independent radiologists. Only features with *ICCs* ≥ 0.75 were retained for subsequent modeling.

Model predictive performance was quantified using standard binary classification metrics, including receiver operating characteristic area under the curve (*ROC-AUC*), accuracy, precision, recall and F1-score, alongside confusion matrix visualization. For internal stratified five-fold cross-validation, all reported metrics represented fold-averaged results. A fixed probability cutoff of 0.5 was uniformly applied to all model outputs to generate confusion matrices and facilitate fair cross-model comparisons, the conventional threshold for binary classification algorithm evaluation. While this hybrid model was developed for clinical auxiliary SDM prediction, the 0.5 cutoff merely served the purpose of model performance comparison; customized thresholds could be selected according to clinical screening priorities in actual diagnostic practice.

Two supplementary analyses were conducted on the optimal MLP multimodal hybrid model validated on the independent external cohort (n=25) to quantify predictive uncertainty and mitigate small-sample bias. We generated 10,000 bootstrap replicates to compute two-sided 95% confidence intervals (*CIs*) for accuracy, recall and *ROC-AUC*. Precision–recall area under the curve (*PR-AUC*) was additionally calculated for external-set evaluation, as this metric delivered more credible performance estimates for small cohorts with mild class imbalance.

## 3 Results

### 3.1 Clinical characteristics of the study population

The internal cohort contained 193 NPC patients (94 with SDM, 99 without SDM), and the independent external test cohort included 25 patients (11 with SDM, 14 without SDM) (**Figure 1**). In the internal cohort, no statistically significant intergroup differences in age, gender, height, weight or fasting blood glucose were observed between SDM-positive and SDM-negative patients (all *p* > 0.05), while SUVmax-T showed a significant intergroup difference (*p* < 0.05). For the external cohort, age, gender, weight and SUVmax-T were comparable between the two groups (all *p* > 0.05). Detailed T/N stage distributions and SDM lesion locations are summarized in **Table 1**.

**Table 1:** Patients’ characteristics.

Characteristics	Internal cohort		p	External validation cohort		p
	SDM (n=94)	No SDM (n=99)		SDM (n=11)	No SDM (n=14)	
age	53.17 ± 12.70	52.47 ± 11.98	0.717	53.27 ± 9.85	51.21 ± 6.42	0.534
Sex (Male/Female)	77/17	81/18	0.985	9/2	12/2	0.792
Height	167.41 ± 8.32	165.96 ± 18.16	0.590	N/A	N/A	N/A
Weight	66.18 ± 12.49	68.52 ± 11.62	0.178	62.55 ± 9.47	70.21 ± 10.58	0.072
blood glucose level (mmol/L)	5.79 ± 0.96	5.74 ± 0.92	0.672	N/A	N/A	N/A
SUVmax-T	15.88 ± 6.31	13.48 ± 4.97	0.004	10.92 ± 6.17	12.85 ± 4.54	0.376
Stage T1 T2 T3 T4 N0 N1 N2 N3	5 14 25 50 0 19 33 42	5 13 25 56 1 21 41 36		1 4 2 4 1 0 7 3	3 4 3 4 0 3 7 4	
Site of metastasis						
One site						
Lung Liver Bone Two sites Lung and Bone Lung and Liver Liver and Bone	15 6 47 4 2 20			3 0 5 0 1 2		

### 3.2 Predictive performance of single-modal and dual-modal DL models

**Figure 3:**
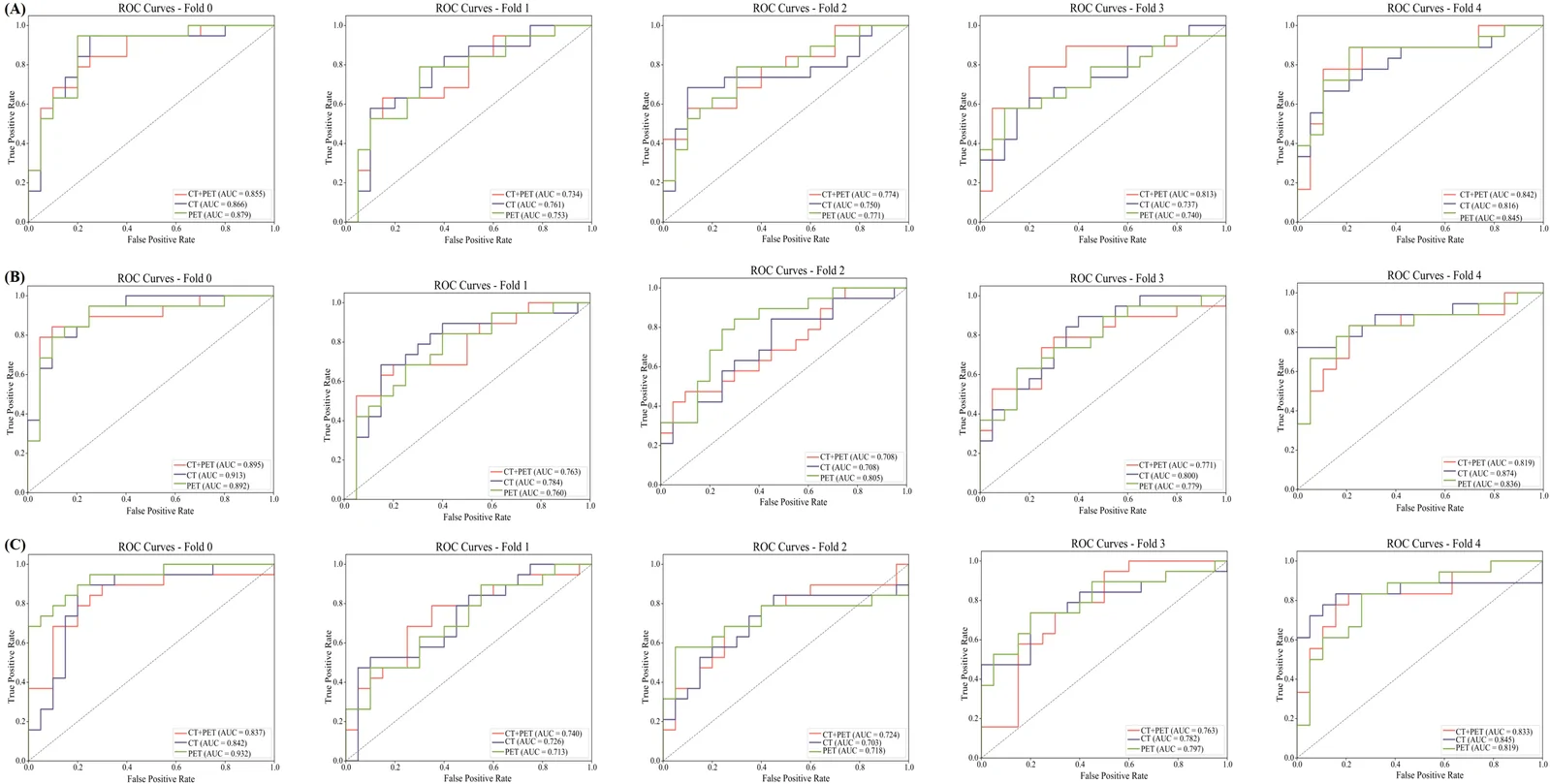
Five-fold cross-validation ROC curves of three CNN architectures trained on CT-only, PET-only, and fused CT+PET images. **(A)** ResNet18 model; **(B)** DenseNet121 model; **(C)** EfficientNet-B0 model.

The dual-modal PET/CT models outperformed single-modality CT-only and PET-only models across all evaluation metrics. Among the three network architectures, ResNet18 achieved the optimal performance under dual-modal input: accuracy = 0.731, F1-score = 0.706, internal *ROC-AUC* = 0.804. Its performance exceeded DenseNet121 (accuracy = 0.710, F1-score = 0.611, *ROC-AUC* = 0.791) and EfficientNet-B0 (accuracy = 0.674, F1-score = 0.664, *ROC-AUC* = 0.779). Detailed performance metrics are listed in **Table 2** and visualized in **Figure 3**.

**Table 2:** Performance of the 9 artificial intelligence prediction models based on CT/PET/CT + PET images.

Models	Accuracy	Precision	Recall	F1-score	ROC-AUC
CT					
Resnet18	0.673	0.823	0.455	0.557	0.786
Densenet121	0.637	0.793	0.484	0.505	0.812
EfficientNet-B0	0.700	0.742	0.693	0.684	0.780
PET					
Resnet18	0.715	0.801	0.614	0.653	0.797
Densenet121	0.678	0.843	0.434	0.557	0.812
EfficientNet-B0	0.674	0.781	0.512	0.585	0.796
CT+PET					
Resnet18	0.731	0.764	0.694	0.706	0.804
Densenet121	0.710	0.872	0.481	0.611	0.791
EfficientNet-B0	0.674	0.711	0.709	0.664	0.779

### 3.3 Predictive performance of multimodal fusion models

The inter-reader reproducibility of deep features, handcrafted radiomic signatures and SUVmax-T was firstly evaluated. All *ICCs* from independent blind delineations by two radiologists exceeded 0.75, demonstrating excellent feature stability for subsequent modeling.

Models relying solely on handcrafted radiomics yielded limited predictive power, with internal AUC ranging from 0.656 to 0.755. Integration of radiomic features substantially improved the performance of DL models (*ROC-AUC*: 0.765–0.824). Further incorporation of clinical variables and SUVmax-T provided additional performance gains, with the optimal *ROC-AUC* reaching 0.839.

**Figure 4:**
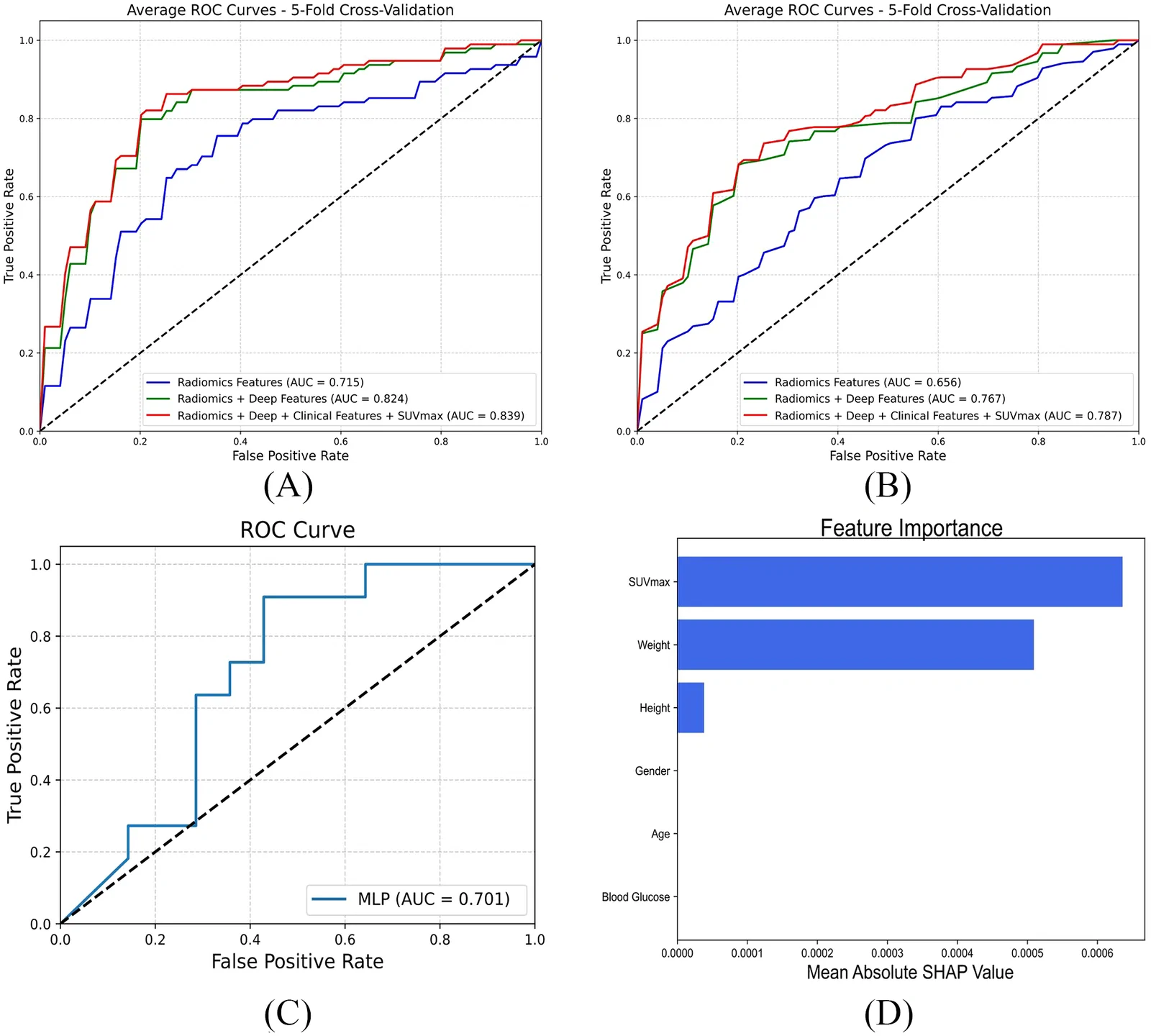
Five-fold cross-validation ROC curve of 3 MLP **(A)** models, 3 RF **(B)** models and MLP-based hybrid model validated on external datasets **(C)**. Results of SHAP analysis **(D)**.

Hybrid models based only on primary tumor radiomic features outperformed models incorporating both primary tumor and CMLNs features. In terms of machine learning frameworks, MLP-based hybrid models achieved better overall predictive performance than RF models. The best-performing MLP hybrid model yielded an internal *ROC-AUC* of 0.839 with a recall of 0.747, whereas the RF model achieved a slightly higher recall of 0.777 but a lower *ROC-AUC* of 0.787 (**Table 3**, **Figure 4**).

**Table 3:** Classification performance of MLP and RF models with distinct multimodal feature combinations in internal cross-validation cohort.

Models	Accuracy	Precision	Recall	F1-score	ROC-AUC
RF					
Radiomics(T)	0.586	0.587	0.543	0.562	0.656
Deep+radiomics(T)	0.712	0.723	0.746	0.713	0.767
Deep+Radiomics(T) +clinical features+ SUVmax-T	0.721	0.733	0.777	0.735	0.787
Radiomics(T+CMLNs)	0.701	0.708	0.650	0.675	0.739
Deep+radiomics(T+CMLNs)	0.727	0.730	0.756	0.732	0.765
Deep+Radiomics(T+CMLNs) +clinical features+ SUVmax-T	0.711	0.717	0.756	0.721	0.752
MLP					
Radiomics(T)	0.694	0.697	0.670	0.683	0.715
Deep+radiomics(T)	0.711	0.709	0.757	0.718	0.824
Deep+Radiomics(T) +clinical features+ SUVmax-T	0.711	0.714	0.747	0.718	0.839
Radiomics(T+CMLNs)	0.680	0.680	0.640	0.657	0.755
Deep+radiomics(T+CMLNs)	0.700	0.709	0.712	0.701	0.777
Deep+Radiomics(T+CMLNs)+clinical features+ SUVmax-T	0.700	0.704	0.713	0.702	0.783

Permutation feature importance analysis based on the RF model identified SUVmax-T as the most dominant predictive feature, surpassing all five clinical covariates (**Table 3**, **Figure 4**).

#### 3.3.1 Incremental value of multimodal imaging signatures over conventional TNM staging

To quantify whether our multimodal model provides additional predictive value beyond routine anatomical staging, a standalone MLP benchmark model was constructed using only categorical T and N stage as one-hot encoded input features. The staging-only model exhibited poor discriminative power for SDM prediction, with an internal *ROC-AUC* of 0.476 and an external *ROC-AUC* of 0.513. Stepwise integration of SUVmax-T metabolic parameters, dual-modal DL features and primary tumor radiomic signatures gradually boosted predictive efficacy. Our final multimodal hybrid model achieved an internal *ROC-AUC* of 0.839 and external *ROC-AUC* of 0.701, both substantially higher than the staging-only benchmark.

Notably, the external T+N+clinical features+SUVmax subgroup attained a recall of 1.000. This subgroup achieved full detection of all SDM cases but generated abundant false-positive predictions, resulting in limited *ROC-AUC* improvement.

These findings further demonstrate that multimodal DL and radiomic biomarkers can comprehensively characterize tumor morphological and glycolytic heterogeneity ignored by morphology-only T/N staging. Therefore, we discarded subjective categorical staging variables and retained fully automated imaging quantitative features to construct the final predictive model with superior discriminative performance.

#### 3.3.2 External validation and bootstrap uncertainty analysis

Independent external validation of the optimal MLP hybrid model was performed using the multicenter external cohort (n = 25). The model achieved an accuracy of 0.680 (95% bootstrap *CIs*: 0.480–0.840), precision of 0.615, recall of 0.727 (95% bootstrap *CIs*: 0.437–1.000), F1-score of 0.667, *ROC-AUC* of 0.701 (95% bootstrap *CIs*: 0.471–0.900), and *PR-AUC* of 0.571 (**Table 4**). All confidence intervals were calculated based on 10,000 bootstrap resampling replicates.

**Table 4:** Performance comparison of integrated models versus baseline T and N staging for SDM prediction in internal and external cohorts.

MLP Models	Features	Accuracy	Precision	Recall	F1-score	ROC-AUC
Internal cohort	T+N	0.514	0.498	0.427	0.458	0.476
	T+N+clinical features+ SUVmax-T	0.486	0.473	0.446	0.458	0.513
	Deep features +T+N	0.658	0.64	0.597	0.58	0.658
	Deep+Radiomics(T)+clinical features+ SUVmax-T	0.711	0.714	0.747	0.718	0.839
External cohort	T+N	0.44	0.364	0.364	0.364	0.513
	T+N+clinical features+ SUVmax-T	0.600	0.524	1.000	0.688	0.636
	Deep features +T+N	0.480	0.458	1.000	0.629	0.688
	Deep+Radiomics(T)+clinical features+ SUVmax-T	0.680	0.615	0.727	0.667	0.701

**Figure 5:**
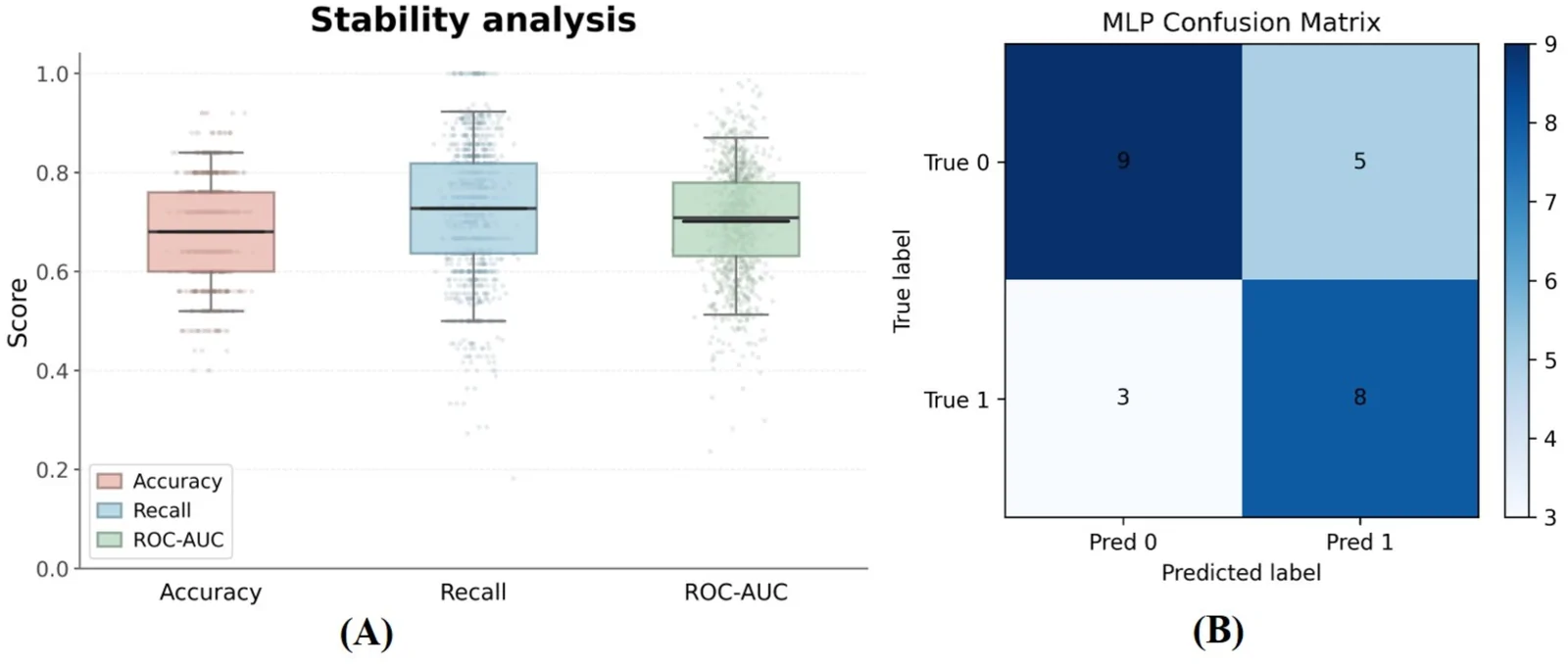
External validation quantitative results of the top-performing MLP multimodal hybrid model on the independent multicenter test set (n = 25, 11 SDM, 14 non-SDM). **(A)** Box plots overlaid with scattered raw values showing the full distribution of bootstrap-derived accuracy, recall, and *ROC-AUC* scores from 10,000 resampling iterations for uncertainty quantification. **(B)** Confusion matrix for SDM classification, generated using a unified prediction probability threshold of 0.5 without threshold re-optimization on external data. True label 0 = non-SDM, True label 1 = SDM.

Box plots illustrating the bootstrap distribution of accuracy, recall and ROC-AUC are shown in **Figure 5A**, and the confusion matrix generated under a fixed classification threshold of 0.5 is displayed in **Figure 5B**.

**Figure 6:**
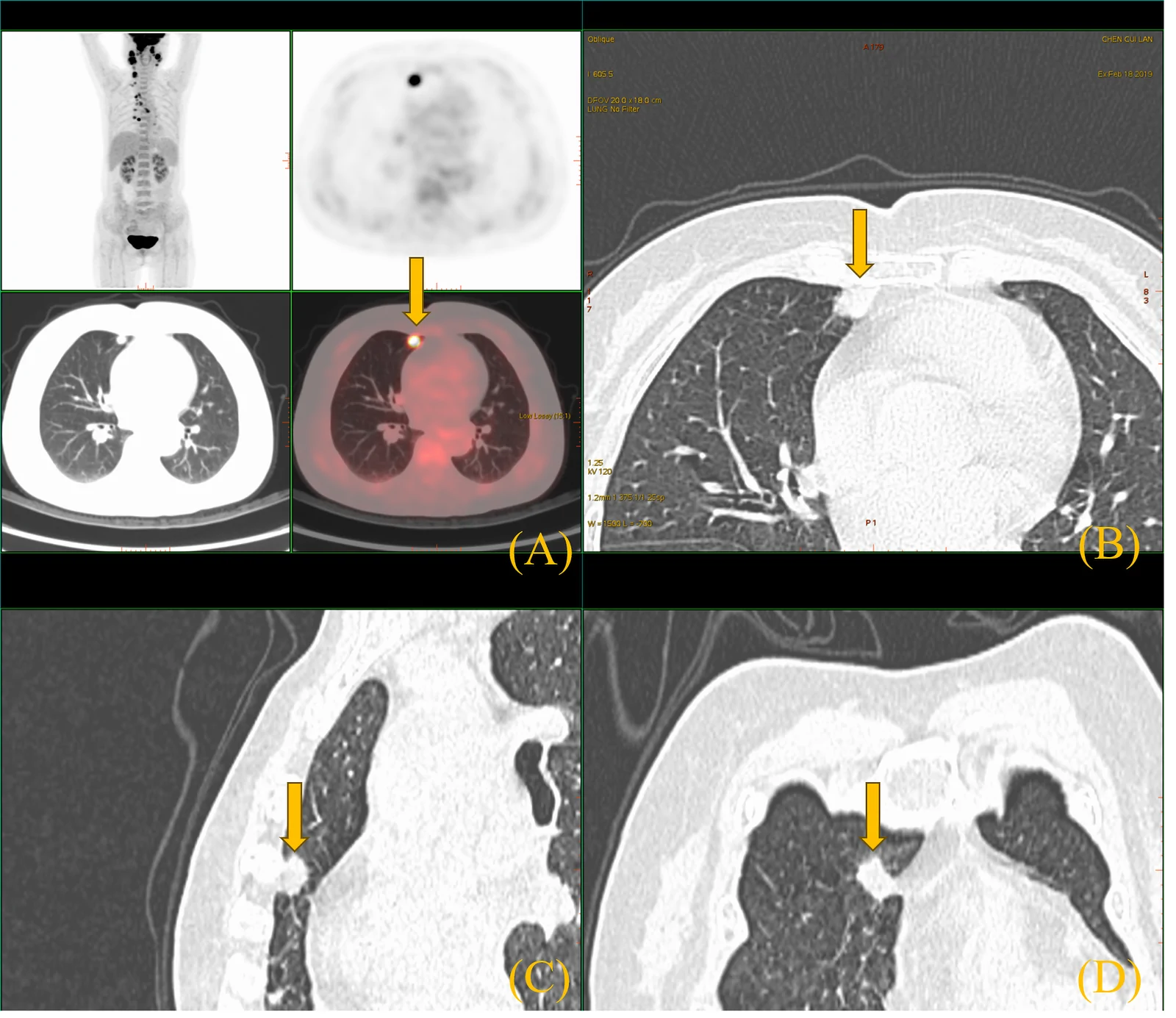
A 64-year-old female patient with undifferentiated carcinoma of the nasopharynx identified via nasopharyngeal endoscopy underwent ¹^8^F-FDG PET-CT **(A)**, which revealed a solitary irregular nodule in the anterior segment of the right upper lung lobe (indicated by the yellow arrow) with increased FDG uptake (SUVmax = 12.38). The lesion showed ill-defined borders with the adjacent mediastinal pleura. Thin-section 1.25 mm CT images in axial **(B)**, sagittal **(C)**, and coronal **(D)** planes demonstrated the irregular morphology of the nodule, which appeared less rounded than typical metastatic lesions. An experienced physician with over 20 years of expertise in ¹^8^F-FDG PET-CT interpretation considered the evidence insufficient to definitively diagnose the nodule as a metastasis at the time of examination. The MLP model (integrating Deep + Radiomics(T) + clinical features + SUVmax) calculated the probability of distant metastasis in this patient as 0.9999999997. The MLP-based hybrid model may serve as auxiliary reference for preliminary risk screening of occult SDM.

**Figure 7:**
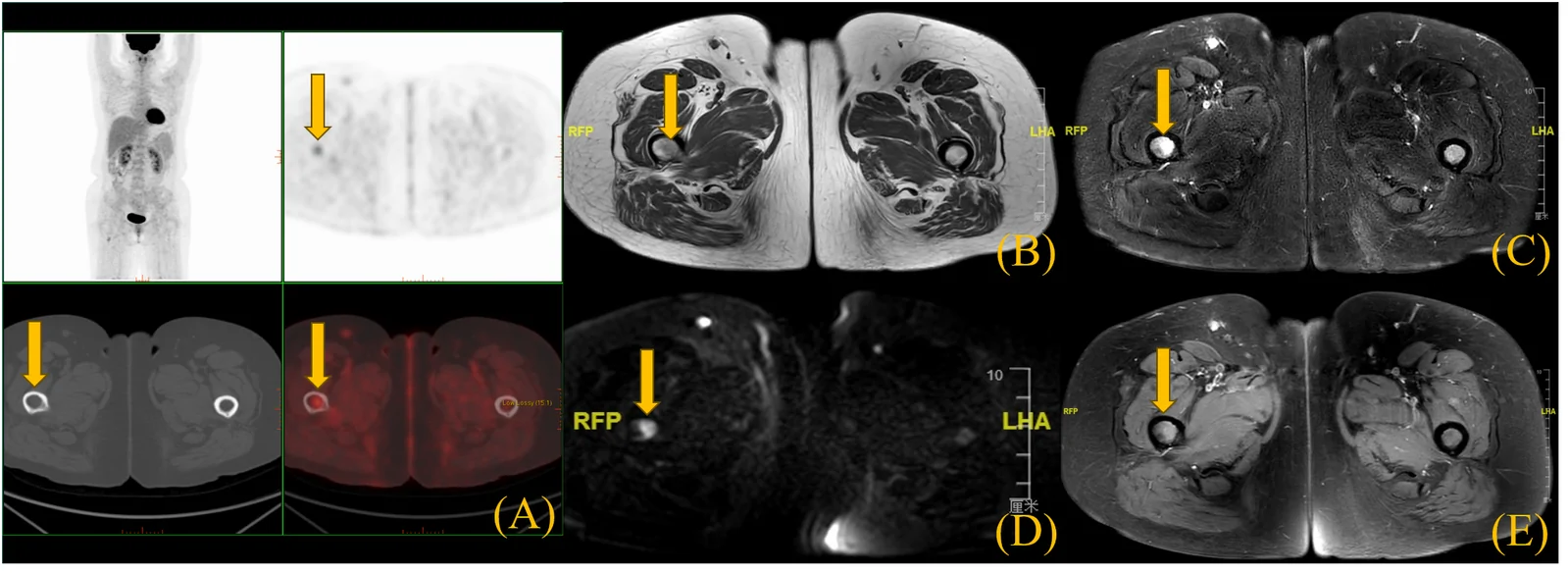
A 51-year-old female patient was diagnosed with poorly differentiated squamous cell carcinoma of the nasopharynx by nasopharyngeal endoscopy. ¹^8^F-FDG PET-CT **(A)** revealed a nodular focus of increased FDG uptake in the bone marrow cavity of the right upper femur with SUVmax = 3.21. The density of the bone marrow cavity was slightly higher compared to the contralateral side. A physician with nearly 20 years of experience in ¹^8^F-FDG PET-CT interpretation considered the lesion likely benign at the time of examination. On T1WI **(B)**, the lesion showed slightly hypointense signal; on T2WI **(C)** and DWI **(D)**, it appeared hyperintense; and mild enhancement was observed after contrast administration **(E)**. Another radiologist with approximately 20 years of imaging experience regarded the lesion as metastatic. The MLP model (Deep + Radiomics(T) + clinical features + SUVmax) calculated the probability of distant metastasis in this patient as 0.9082629675, further supporting that our model provides auxiliary reference for preliminary risk screening of SDM.

To further visualize the clinical applicability of the proposed model for identifying occult SDM, two representative NPC cases with discrepant risk stratification results were presented in **Figure 6** and **Figure 7**.

## 4 Discussion

The primary objective of this study was to construct a DL predictive model for pretreatment SDM risk stratification in NPC based on bimodal ¹^8^F-FDG PET/CT scans. Across all evaluation metrics, dual-modal PET/CT DL models outperformed single-modality CT-only and PET-only networks. The ResNet18 bimodal framework achieved optimal performance (accuracy = 0.731, F1-score = 0.706, *ROC-AUC* = 0.804), surpassing the DenseNet121 (accuracy = 0.710, F1-score = 0.611, *ROC-AUC* = 0.791) and EfficientNet-B0 (accuracy = 0.674, F1-score = 0.664, *ROC-AUC* = 0.779) counterparts. This superior performance suggests ResNet18 can effectively capture complementary anatomical and metabolic information from fused PET/CT data.

Our secondary research aim was to explore whether handcrafted radiomic signatures and metabolic biomarkers could further boost the discriminative capacity of DL pipelines. Models constructed solely from radiomic features yielded limited predictive power, with internal *ROC-AUC* ranging only from 0.656 to 0.755. Stepwise integration of radiomic features, clinical covariates and SUVmax-T brought steady performance improvements, lifting the upper bound of *ROC-AUC* to 0.839. Among all multimodal hybrid models, the MLP pipeline reached the highest AUC of 0.839 (recall = 0.747), whereas RF offered slightly higher recall (0.777) but inferior overall discrimination (*ROC-AUC* = 0.787). Permutation feature importance analysis further demonstrated that SUVmax-T was the top predictive feature across all five clinical covariates in the RF model.

Benchmark comparison against T/N staging alone validated the extra predictive value of our imaging signatures. Models built purely on anatomical T and N stages exhibited poor SDM discrimination, because manual TN evaluation suffers from radiologist subjectivity and inter-reader inconsistency. Our final hybrid model only adopts automatically extracted objective biomarkers including deep features, radiomics and SUVmax-T. Its markedly higher internal and external *ROC-AUC* demonstrates that quantitative PET/CT metrics reflect intratumoral morphological and glycolytic heterogeneity missed by conventional staging, adding unique predictive evidence for detecting occult baseline SDM. Consistent with the inherent generalization challenge of small retrospective cohorts, the MLP hybrid model exhibited reduced predictive performance when tested on the independent external multicenter dataset.

ResNet-18 (40), a residual CNN, alleviates vanishing gradient limitations in deep architectures by incorporating skip connections. Its 18-layer design provides computational efficiency while maintaining robust feature extraction capabilities, rendering it particularly suitable for medical diagnostic imaging tasks with limited sample sizes. This architecture has been adopted in previous NPC prediction studies focusing on post-radiotherapy MDM rather than pretreatment SDM (38).

Both MDM and SDM prediction studies face the challenge of scarce metastatic-positive patients, yet the root causes differ. SDM cases are restricted by the low baseline metastasis prevalence at initial diagnosis, while MDM positive samples rely on long-term follow-up accumulation and still show severe subgroup imbalance in published work. Basic translational research exploring SDM-related molecular biomarkers also only contained very limited numbers of SDM-positive tissue specimens for immunohistochemistry validation (24, 25).

Although the cohort in our study (total n=218) was relatively small, an earlier relevant study with a larger overall sample suffered severe imbalance between MDM and non-MDM subgroups (114 vs 327) (12). Notably, all existing predictive models reported in these references targeted MDM occurring after radiotherapy, rather than pretreatment SDM assessed in our research. To mitigate potential model bias arising from the disparity in sample sizes between SDM-positive and negative patients, we randomly selected 99 non-SDM patients from a pool of 1347 eligible candidates to achieve balanced subgroup distribution, and further adopted stratified five-fold cross-validation and bootstrap resampling to stabilize model evaluation under limited positive samples.

Radiomics improves preoperative diagnostic, predictive, and prognostic precision in NPC (31). However, most radiomics-based predictive studies for NPC focus on post-treatment MDM, while models for pretreatment SDM remain limited. Zhang et al. (13) built an MRI radiomic model combined with N-stage stratification to predict post-radiotherapy MDM risk. Handcrafted radiomics relies on manually predefined feature sets, whereas DL adopts an end-to-end pipeline to automatically extract hierarchical features from raw medical images. In their subsequent work (12), the same group integrated DL and radiomic signatures for NPC MDM prediction and reported a counterintuitive observation: fusion models yielded inferior *ROC-AUC* compared with single-modality DL or radiomics alone. Distinct from their single-modal MRI framework designed for post-treatment MDM prediction, our dual-modal PET/CT model targets pretreatment occult SDM and achieves steady performance improvement via multimodal feature fusion.

Our study also identified an unexpected observation: hybrid models incorporating radiomic features from both primary tumors and CMLNs exhibited inferior performance compared with models using only primary tumor radiomic features. This phenomenon may be attributed to two key factors: the high intrinsic heterogeneity of CMLN imaging signatures and the inherently small volume of metastatic lymph nodes. Both characteristics introduce excessive noise that outweighs valuable predictive signals during multimodal feature fusion. In addition, hybrid models constructed with the MLP algorithm outperformed those using the RF algorithm. As a hierarchical artificial neural network composed of input, multiple hidden, and output layers, the MLP has three core strengths for medical image analysis compared with traditional machine learning algorithms: (a) nonlinear adaptive capability for robust modeling of complex heterogeneous clinical correlations; (b) automated extraction of high-level features directly from raw imaging data; (c) intrinsic parallel processing that speeds up training and inference (44, 45). Consistent with previous evidence that MLP exhibits superior prognostic stratification performance for distant metastasis in uveal melanoma (46), our MLP-based hybrid model showed stronger discriminative power for distinguishing SDM-positive and SDM-negative cases, whereas the RF model presented a slight advantage in true-positive identification. These distinct characteristics support task-oriented model selection: the MLP hybrid model is suitable for general pretreatment risk screening with balanced overall performance, while the RF model is more applicable for high-sensitivity screening of high-risk NPC subgroups.

Permutation feature importance analysis revealed SUVmax-T as the predominant predictive contributor in the RF-based hybrid model, surpassing all five enrolled clinical variables. Consistent with our statistical analysis showing significant SUVmax-T differences between SDM and non-SDM groups, SUVmax-T demonstrated robust predictive potential for baseline SDM risk. As the peak tracer uptake value on PET images, SUVmax-T quantitatively reflects intratumoral glycolytic metabolism and remains the most widely adopted semi-quantitative metabolic biomarker in clinical PET evaluation (27, 47). Feng et al. (27) previously constructed a multimodal PET/MRI signature incorporating SUVmax and total lesion glycolysis to refine pretreatment TNM stratification in NPC, further validating the critical value of primary tumor metabolic biomarkers for individualized NPC risk evaluation.

Given the small, slightly imbalanced external cohort, we adopted both *ROC-AUC* and *PR-AUC* for unbiased model evaluation. The hybrid model achieved an external *ROC-AUC* of 0.701, indicating moderate overall discriminative power. *ROC-AUC* tends to produce overoptimistic estimates on small imbalanced datasets, whereas *PR-AUC* prioritizes correct identification of SDM-positive patients, matching our clinical goal of pretreatment risk screening. The modest external *PR-AUC* of 0.571 reflects unstable prediction stemming from limited sample size and cross-scanner imaging heterogeneity, explaining the performance drop from internal cross-validation to external testing. Taken together, dual-metric evaluation demonstrates promising internal performance, yet robust clinical translation requires validation in larger standardized prospective multicenter cohorts.

## 5 Limitations

This study has several inherent limitations.

First, the internal dataset was retrospectively collected from a single center, which may introduce selection bias even after stratified matching to balance SDM-positive and SDM-negative subgroups.

Second, external validation relied on a small single-center cohort with different PET/CT acquisition protocols; residual inter-scanner domain heterogeneity impaired generalization despite uniform preprocessing.

Third, high-dimensional imaging features raised dimensionality-sample mismatch risks, and overfitting cannot be fully eliminated under limited samples even with ICC filtering and five-fold cross-validation. This constraint stems from the inherently low proportion of SDM-positive patients among newly diagnosed NPC cases, a common challenge across all relevant translational studies.

Fourth, this imaging-focused model excluded EBV-DNA and pathological markers, as standardized, complete laboratory and pathological records were unavailable across our retrospective dual-center cohort.

## 6 Future perspectives

Several actionable optimization strategies are proposed to resolve the above limitations and elevate the robustness and translational value of this SDM prediction model for subsequent research.

First, we will recruit prospective multicenter cohorts with unified imaging and follow-up protocols. This design can lower retrospective selection bias and improve the generalizability of study populations.

Second, domain adaptation algorithms will be introduced to mitigate inter-scanner image heterogeneity and stabilize prediction performance across different medical centers.

Third, multi-stage feature screening and network lightweight optimization will be carried out. These operations cut redundant high-dimensional features and ease overfitting caused by the mismatch between model complexity and limited sample size. Small-sample learning tools such as data augmentation and transfer learning will also be adopted to raise feature extraction efficiency.

Finally, serological and pathological prognostic markers will be incorporated into the multimodal framework. Combined multi-source biomarkers can realize more complete risk stratification and refine individualized pretreatment assessment of occult SDM in NPC patients.

## 7 Conclusions

This preliminary exploratory study constructed a multimodal prediction model based on pretreatment ¹^8^F-FDG PET/CT primary tumor and cervical lymph nodes images to non-invasively predict SDM in NPC. Fusion of ResNet18 deep features, primary tumor radiomics signatures and SUVmax-T further elevated model discrimination ability, and the multimodal hybrid model achieved superior performance compared with simple anatomical T and N staging. This pipeline can serve as an auxiliary preoperative risk stratification tool to identify high-risk patients with occult SDM, and provides a solid foundation for future optimized artificial intelligence prediction models for NPC metastasis.

## Data Availability

The raw data supporting the conclusions of this article will be made available by the authors, without undue reservation.
